# Retraction: DNA Damage, Homology-Directed Repair, and DNA Methylation

**DOI:** 10.1371/journal.pgen.1011921

**Published:** 2025-10-27

**Authors:** The PLOS Genetics Editors

After this article [1] and Correction [2] were published, additional concerns were raised regarding Figs 1 and 5. Specifically:

- Lanes 1 and 2 of the Fig 1Bb right GFP panel appear similar.
- The following Fig 5B panels appear more similar than would be expected from independent results:
  - The WT + I-Scel panel and the WT + I-Scel 5-AzadC panel
  - The Dnmt1-/- + I-Scel panel and the Dnmt1-/- + I-Scel 5-AzadC panel

The corresponding author (EVA) disagrees with the concerns pertaining to Fig 1B. They stated that the original uncropped images underlying this figure are no longer available.

In response to the concerns regarding Fig 5B, the corresponding author (EVA) stated that it is likely that some of the dots may be similar, because the processor used to analyze the files was a 8bit fcs files analyzer which converted a collection of 100K points into a 256x256 dot plot. They provided original flow cytometry data files for Fig 5B and individual-level quantitative data for Figs 5A and 5C. They also shared screenshots of their own reanalysis of some of the original and replicate data files, but details regarding the gating windows used to construct the published panels were not available.

PLOS consulted an independent expert who assessed the original files underlying the FACS plots in Fig 5B and the reanalysis of some original files completed by the authors. The independent expert stated that these original files were generated on two different dates using two different flow cytometer instruments. They advised that the flow cytometry dot plots for WT and Dnmt-/- cells in both the control and + I-Scel 5-AzadC groups in Fig 5B did not accurately represent the corresponding underlying data files, and the data provided by the authors were considered insufficient to resolve the concerns raised with Fig 5B.

In light of the above unresolved concerns which question the integrity and reliability of these data, the *PLOS Genetics* Editors retract this article.

AP, TA, BL, SM, RI, AF, MRS, MTM, LC, MEG, and EVA did not agree with the retraction. CC, AM, and ADP either did not respond directly or could not be reached.
